# Laparoscopic Cholecystectomy for a Patient after Percutaneous Endoscopic Gastrostomy due to Myotonic Dystrophy: A Case Report and Literature Review

**DOI:** 10.70352/scrj.cr.25-0038

**Published:** 2025-04-01

**Authors:** Kei Naito, Takanori Konishi, Tsukasa Takayashiki, Shigetsugu Takano, Daisuke Suzuki, Nozomu Sakai, Isamu Hosokawa, Takashi Mishima, Hitoe Nishino, Kensuke Suzuki, Shinichiro Nakada, Masayuki Ohtsuka

**Affiliations:** Department of General Surgery, Graduate School of Medicine, Chiba University, Chiba, Chiba, Japan

**Keywords:** laparoscopic cholecystectomy, gastrostomy, percutaneous endoscopic gastrostomy, myotonic dystrophy

## Abstract

**INTRODUCTION:**

Percutaneous endoscopic gastrostomy (PEG) is commonly performed for enteral nutrition in patients with various diseases. However, there are few reports on abdominal surgeries for patients after PEG, and the tips for these procedures have not been established. Specifically, in laparoscopic surgeries of the upper abdomen, a gastrostomy can interfere with the surgical field. In addition, perioperative management of concomitant diseases that require PEG placement, including neuromuscular disorders, is required.

**CASE PRESENTATION:**

A 64-year-old man with a PEG due to malnutrition from myotonic dystrophy was diagnosed with acute cholangitis and choledocholithiasis. After lithotomy during endoscopic retrograde cholangiopancreatography, the patient was scheduled for laparoscopic cholecystectomy for the cholelithiasis. Although the patient had myotonic dystrophy and limited respiratory function, his general condition was deemed acceptable for surgery. Given the potential risk of gastrostomy injury and the need to ensure sufficient working space, the location of the gastrostomy tube was preoperatively confirmed via a computed tomography scan, and precautions were taken to prevent injuries caused by port insertion, forceps manipulation, and pneumoperitoneum during the procedure. Ultimately, the gastrostomy did not interfere with manipulation around the gallbladder, and the surgery was completed without any complications. To manage myotonic dystrophy, general intravenous anesthesia with propofol was administered, with minimal use of muscle relaxants during surgery. Postoperatively, the patient was managed with high nasal flow to reduce respiratory workload, epidural anesthesia to prevent respiratory depression due to pain, and early initiation of aggressive physical therapy. The patient was discharged on postoperative day 4 without complications.

**CONCLUSIONS:**

Using appropriate surgical strategies, laparoscopic cholecystectomy may be safely performed for patients with myotonic dystrophy after PEG.

## Abbreviations

CT
: computed tomography
PEG
: percutaneous endoscopic gastrostomy
VC
: vital capacity

## INTRODUCTION

Although percutaneous endoscopic gastrostomy (PEG) has been commonly performed,^1)^ reports on abdominal surgeries for patients after PEG remain limited, and the safety of these procedures has not been adequately discussed. Specifically, in laparoscopic surgery of the upper abdomen, the presence of a gastrostomy can interfere with the surgical field, which may influence standard surgical techniques. In addition, perioperative management of concomitant diseases that require PEG placement, including neuromuscular disorders, is required. Here, we present our experience with laparoscopic cholecystectomy in a patient with myotonic dystrophy who had previously undergone PEG placement.

## CASE PRESENTATION

A 64-year-old man with myotonic dystrophy and PEG placement was referred to our hospital with fever and abdominal pain. Contrast-enhanced computed tomography (CT) revealed gallbladder and common bile duct stones. The patient was diagnosed with acute cholangitis caused by common bile duct stones. For treatment, lithotomy via endoscopic retrograde cholangiopancreatography was performed, and antibiotics were administered. After discharge, he was scheduled to undergo cholecystectomy for cholelithiasis to prevent recurrence of acute cholangitis.

**Fig. 1:**
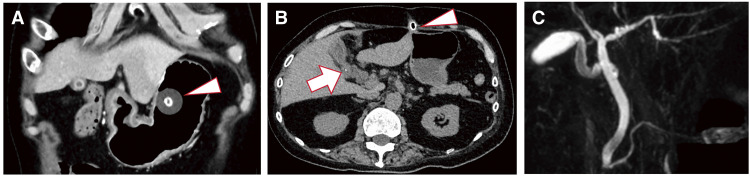
(**A** and **B**) Preoperative CT showed a balloon-type gastrostomy in the upper body of the stomach (arrowhead) and a gallbladder stone (arrow). (**C**) Magnetic resonance cholangiopancreatography showed no morphological abnormalities of the bile duct bifurcation. CT, computed tomography.

The patient developed myotonic dystrophy at the age of 51 years, presenting with level 4 muscle weakness on manual muscle testing in all 4 limbs and the trunk. At the age of 59 years, the patient underwent PEG placement in the epigastric region for enteral nutrition. The patient was classified as American Society of Anesthesiologists physical status class 3, with a height of 163 cm, a weight of 48.5 kg, and a body mass index of 18.3 kg/m^2^. Preoperative blood tests revealed no abnormalities that could interfere with general anesthesia. Cardiac ultrasonography revealed a normal ejection fraction of 64% and no significant valvular disease. Spirometry indicated restricted ventilatory impairment, with a vital capacity (VC) of 2.33 L and a %VC of 64.2%. The patient demonstrated exercise tolerance of greater than 4 metabolic equivalents. Preoperative CT revealed balloon-type gastrostomy of the upper body of the stomach. The gallbladder was atrophied, and gallstones were observed (**Fig. 1A** and **1B**). Magnetic resonance cholangiopancreatography revealed no morphological abnormalities at the bile duct bifurcation (**Fig. 1C**).

Preoperative examination revealed limited respiratory function, but the patient’s general condition was deemed acceptable for surgery. A laparoscopic cholecystectomy was scheduled for the patient. Given the history of myotonic dystrophy, we planned to administer general intravenous anesthesia using propofol with minimal administration of muscle relaxants to reduce the risk of complications.

**Fig. 2:**
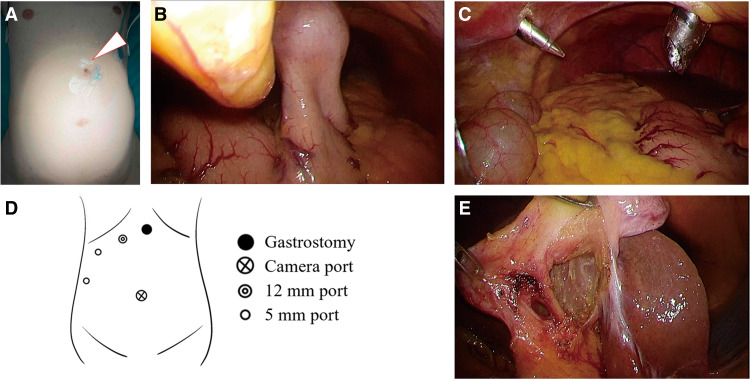
(**A**) The gastrostomy was placed in the epigastric region. (**B**) The gastrostomy was identified on the left side of the hepatic round ligament, and the gastric body was suspended so that it was attached to the abdominal wall. (**C** and **D**) We used an American-style 4-port technique with the port placement shifted slightly to the right side of the usual position. (**E**) The critical view of safety was identified.

The patient underwent laparoscopic cholecystectomy under general and epidural anesthesia in the supine position. The epidural catheter was inserted while the patient was awake. The gastrostomy tube was covered and protected with a film (**Fig. 2A**). As the gastrostomy tube was placed in the upper part of the stomach, a camera port was inserted through the umbilicus. Pneumoperitoneum was initiated at a low pressure, with the insufflation pressure gradually increased while confirming the working space. After confirmation of fistula integrity and maintenance of adequate working space, the intra-abdominal pressure was maintained at a standard level of 10 mmHg. Upon inspection of the abdominal cavity, a gastrostomy was identified on the left side of the hepatic round ligament, with the gastric body attached to the abdominal wall (**Fig. 2B**). The gastrostomy did not interfere with manipulation around the gallbladder, and sufficient working space was achieved with minimal use of muscle relaxants. Laparoscopic port placement was carefully planned to avoid injury during port insertion or forceps manipulation. We used the American-style 4-port technique, with the port placement shifted approximately 3–4 cm to the right of the usual position (**Fig. 2C** and **2D**). This arrangement effectively prevented damage to the gastrostomy tube during port insertion and ensured that the lifted stomach did not interfere with the line connecting the gallbladder and forceps, thereby minimizing the risk of injury from forceps manipulation. Despite the port placement being more rightward than usual, there were no issues with manipulation around the gallbladder. Although the gallbladder wall thickness was minimal, the surgical procedure proceeded relatively smoothly, and the critical view of safety was achieved (**Fig. 2E**). The cystic artery and duct were clipped and divided, and the gallbladder was removed. Regarding the anesthesia, rocuronium was given in a dose of 10 mg during induction of anesthesia, and 3 additional doses of 5 mg each during surgery, which was a lower dose of muscle relaxants than usual. Intraoperatively, we confirmed that there was no evident injury to the gastrostomy site, and the surgery was completed. The pathological examination revealed chronic cholecystitis.

Postoperatively, the patient was extubated and managed with nasal high flow to reduce respiratory workload. Appropriate pain management, including epidural anesthesia, also contributes to alleviating the risk of pain-induced respiratory depression. Early initiation of aggressive physical therapy effectively prevented any decline in activities of daily living. The patient was discharged on postoperative day 4 without complications.

## DISCUSSION

In 1980, Gauderer et al. introduced a PEG procedure that did not require general anesthesia.^2)^ Recently, gastrostomies have been shown to play an important role as a route for nutritional administration. PEG is used to treat malnutrition due to various diseases, such as neuromuscular diseases; esophageal, head, and neck malignancies; pneumonia; dementia; and psychiatric disorders.^1)^ In Japan, the annual number of PEG insertions is reported to have increased to 96000–119000,^1)^ with a higher prevalence than that in Western countries.^3)^ In a study of 3756 patients with neuromuscular disease, Hanai et al. reported that 325 patients (8.7%) underwent PEG,^4)^ suggesting the potential benefits of this procedure for many patients.

The general incidence of cholelithiasis is relatively high, affecting 15% of the population of the United States.^5)^ Especially in patients who undergo gastrostomy, the occurrence of cholelithiasis is increased because of inadequate gallbladder contraction caused by enteral nutrition, leading to bile stasis.^6)^ In addition, the risk of severe cholecystitis or cholangitis caused by gallbladder stones may be higher in these patients, as patients with neuromuscular diseases sometimes have difficulty expressing their symptoms.^6)^ The mortality rate of patients after PEG has been reported to be relatively high, reaching 59.2% 2 years after gastrostomy tube insertion.^7)^ However, advances in ventilation technology and gene therapy have significantly improved the survival of patients with neuromuscular diseases.^8,9)^ While a gastrostomy may obstruct the surgical field, PEG placement itself is not a contraindication for surgery, provided that the patient’s condition is appropriately assessed. In this case, the patient had muscular dystrophy, a refractory neuromuscular disease. However, surgery was performed because the patient’s condition was expected to have a good long-term prognosis, and there was a risk of recurrent cholecystitis or cholangitis due to cholelithiasis.

Reports of abdominal surgeries, particularly laparoscopic surgeries, in patients with a gastrostomy and cholelithiasis remain rare, with only 1 case reported in the PubMed database.^10)^ Therefore, the safety and tips of these procedures have not been adequately described. We have developed several detailed surgical strategies for laparoscopic cholecystectomy in patients after PEG. The most critical aspect of a surgical procedure is preventing injury to the gastrostomy site. There are 3 potential types of gastrostomy injuries: port insertion, forceps manipulation, and pneumoperitoneum. To minimize these risks, the ports were carefully placed away from the gastrostomy site. In addition, ports were positioned to avoid interference from an elevated stomach along the line connecting the gallbladder and forceps. In particular, it may be critical to proceed with caution when a gastrostomy is positioned near the gastric pylorus. American- and French-style port placements are the 2 common approaches for laparoscopic cholecystectomy. The choice of port placement should be selected based on the site of gastrostomy to minimize its impact on the procedure. In this case, gastrostomy was located in the upper part of the stomach, positioned to the left of the hepatic round ligament. Therefore, we adopted the American-style 4-port technique to prevent interference with the operation. The condition of the intraperitoneal cavity after PEG cannot be directly visualized before surgery. Therefore, to ensure safe port placement, it is necessary to identify the position of the gastrostomy tube through physical examination and CT preoperatively. Moreover, it is important to confirm the absence of gastrostomy tube injury during surgery, specifically prior to port removal. Surace et al.^10)^ pointed out the possibility of gastrostomy injury due to pneumoperitoneum during laparoscopic surgery. Gastrostomy tube fistulas generally mature within 1–2 weeks; however, maturation may be delayed to 3–4 weeks in patients receiving corticosteroids, those who are malnourished, or those with ascites or other conditions.^11)^ Therefore, replacing the gastrostomy tube should be avoided within the first 4 weeks, as an immature fistula increases the risk of peritonitis due to leakage of gastric contents into the peritoneal cavity.^12)^ Based on these findings, it is essential to recognize the date of PEG tube placement, and surgery should be avoided in the early stages after PEG.

Another critical aspect of surgery for patients after PEG is the consideration of underlying diseases that necessitate gastrostomy. It is essential to evaluate whether a patient can tolerate general anesthesia. In addition, careful perioperative management, including consideration of anesthetic dosage limitations, is crucial. Due to the increased sensitivity to muscle relaxants and the potential risk of muscle relaxant antagonists inducing myotonia in myotonic dystrophy,^13)^ the use of these medications was minimized during anesthesia in this case. In addition, because inhaled anesthetics may impair ventilation and reduce cardiac function through muscle rigidity in myotonic dystrophy,^14)^ a general intravenous anesthetic with propofol was administered. Because patients with neuromuscular diseases are at a high risk of developing postoperative respiratory complications,^14)^ postoperative respiratory support, including epidural anesthesia for postoperative pain relief, nasal high flow after extubation, and early initiation of aggressive physical therapy, should be performed. A previous systematic review indicates that epidural anesthesia is not recommended for standard laparoscopic cholecystectomy in terms of postoperative analgesia due to the risk of complications and the potential for failure of the anesthetic technique.^15)^ However, epidural anesthesia also provides intraoperative analgesia and muscle relaxation through sympathetic blockade. We chose epidural anesthesia not only for its postoperative analgesic benefits but also for its potential to reduce the use of intraoperative muscle relaxants. Laparoscopic surgery was selected as the surgical technique for this case. Laparoscopic surgery has been reported to be less invasive than open surgery and has been shown to reduce the risk of respiratory complications.^16,17)^ Nevertheless, if a gastrostomy interferes with the view around the gallbladder and laparoscopic manipulation is deemed excessively risky, open surgery should be considered.

## CONCLUSIONS

Laparoscopic cholecystectomy may be safely performed for patients after PEG due to myotonic dystrophy with meticulous surgical planning, including the prevention of gastrostomy injury and perioperative management of the underlying disease.

## DECLARATIONS

### Funding

None to declare.

### Authors’ contributions

The authors confirm their contributions to the paper as follows: study conception and design, KN and TK; data collection, KN; analysis and interpretation of results, KN and TK. MO provided final approval for this version of the manuscript. All the authors have read and approved the final version of the manuscript.

### Availability of data and materials

The data supporting the findings of this study are available from the corresponding author upon reasonable request.

### Ethics approval and consent to participate

Ethics approval is not applicable because this is a case report.

### Consent for publication

Informed consent to publish has been obtained.

### Competing interests

The authors declare that they have no competing interests.
